# Sea Cucumber Hydrolysate Alleviates Immunosuppression and Gut Microbiota Imbalance Induced by Cyclophosphamide in Balb/c Mice through the NF-κB Pathway

**DOI:** 10.3390/foods12081604

**Published:** 2023-04-10

**Authors:** Jing Mao, Shunqin Li, RongRong Fu, Yijin Wang, Jing Meng, Yan Jin, Tao Wu, Min Zhang

**Affiliations:** 1 State Key Laboratory of Food Nutrition and Safety, Food Biotechnology Engineering Research Center of Ministry of Education, College of Food Science and Engineering, Tianjin University of Science & Technology, Tianjin 300457, China; 2 China-Russia Agricultural Processing Joint Laboratory, Tianjin Agricultural University, Tianjin 300384, China

**Keywords:** sea cucumber hydrolysate, cyclophosphamide, NF-κB, gut microbiota

## Abstract

This study aimed to investigate the effect of sea cucumber hydrolysate (SCH) on immunosuppressed mice induced by cyclophosphamide (Cy). Our findings demonstrated that SCH could increase the thymus index and spleen index, decrease the serum alanine transaminase (ALT) and aspartate aminotransferase (AST) levels, increase the serum IgG and small intestinal sIgA levels, reduce small intestinal and colon tissue damage, and activate the nuclear factor-κB (NF-κB) pathway by increasing TRAF6 and IRAK1 protein levels, as well as the phosphorylation levels of IκBα and p65, thereby enhancing immunity. In addition, SCH alleviated the imbalance of the gut microbiota by altering the composition of the gut microbiota in immunosuppressed mice. At the genus level, when compared with the model group, the relative abundance of *Dubosiella*, *Lachnospiraceae*, and *Ligilactobacillus* increased, while that of *Lactobacillus*, *Bacteroides*, and *Turicibacter* decreased in the SCH groups. Moreover, 26 potential bioactive peptides were identified by oligopeptide sequencing and bioactivity prediction. This study’s findings thus provide an experimental basis for further development of SCH as a nutritional supplement to alleviate immunosuppression induced by Cy as well as provides a new idea for alleviating intestinal damage induced by Cy.

## 1. Introduction

The immune system is a network composed of cells, tissues, and organs that functions to eliminate potentially harmful substances from the body [1]. The gut is the body’s largest digestive organ, and the intestinal mucosa is the first line of defense against pathogenic and non-pathogenic microorganisms [2]. The trillions of bacteria that colonize the mammalian gut are called gut microbiota [3]. Gut microbiota and its metabolites play an important role in the immune system’s functions, which is to maintain the symbiotic relationship between the host and microorganisms [4,5,6]. Gut microbiota also protects the host by promoting immune homeostasis, immune response, and preventing pathogen colonization [3].

Cyclophosphamide (Cy) belongs to the mustard alkylating agent oxazaphosphorine family and was first synthesized by Norbert Brock in 1958 [7]. Cy is an effective chemotherapy agent used in the treatment of lymphoma and breast and ovarian cancers; it is also used as an immunosuppressant in bone marrow transplantation [8]. Currently, high doses of Cy (>120 mg/kg) are used as immunosuppressants for the treatment of autoimmune diseases such as lupus, while low doses (1−3 mg/kg) are used in cancer treatment [7]. Cy can reduce the number of white blood cells and the activity of lymphocytes in the spleen [9]. Long−term exposure to Cy can inhibit the activation, proliferation, and differentiation of B cells as well as reduce serum antibody levels [10]. The intraperitoneal injection of Cy can cause weight loss, immune organ index reduction, cellular immune factors, immunoglobulin reduction, and immune cell activity reduction in mice [11,12]. Cy can also modify the gut microbiota composition and destroy the intestinal mucosa of mice [13,14].

Sea cucumbers have long been used in the food and medicine industries in Asian countries. Past studies have demonstrated that sea cucumber peptide possesses antioxidative, hypoglycemic, memory-enhancing, and other biological activities [15,16,17]. Moreover, sea cucumber oligopeptides have been reported to exert immune activation on RAW264.7 cells through MAPK and the nuclear factor−κB (NF−κB) pathways [18]. However, the effect of sea cucumber hydrolysate (SCH) on intestinal immunity has rarely been reported. In this study, Cy was used to establish an immunosuppressive mouse model. The effect of SCH on Cy-induced immunosuppressed mice was investigated by determining the immune organ index, serum, and intestinal tissue biochemical index, small intestinal and colon tissue damage, the expression level of the NF-κB pathway-related protein, and gut microbiota.

## 2. Materials and Methods

### 2.1. Materials and Reagents

Alanine transaminase (ALT) and aspartate aminotransferase (AST) kits were purchased from Nanjing Jiancheng Bioengineering Research Institute Co., Ltd. (Nanjing, China). Immunoglobulin G (IgG) and secretory immunoglobulin A (sIgA) detection kits were purchased from Shanghai Enzyme Linked Biotechnology Co., Ltd.(Shanghai, China). The primary antibodies used in this study were as follows: anti−p−IκBα (1:1000, bs−2513R, Bioss (Beijng, China)), anti−IκBα (1:1000, bs−1287R, Bioss), β−actin (1:2000, 20536−1−AP, Proteintech (Wuhan, China)), anti−p65 (1:1000, 10745−1−AP, Proteintech), anti−TRAF6 (1:5000, 66498−1−Ig, Proteintech), anti−IRAK1 (1:1000, 10478−2−AP, Proteintech), and anti−p−p65 (1:500, AP3749a, Abcepta (Suzhou, China)). Among the primary antibodies used in Western blotting, only anti-TRAF6 (clone number: 1D1E1) was a murine monoclonal antibody, while the remaining were rabbit polyclonal antibodies.

### 2.2. Preparation of SCH

SCH was derived from sea cucumber (*Acaudina molpadioides*) via enzymatic hydrolysis and produced by Bestlife Biotechnology Co., Ltd. (Tangshan, China). Briefly, the sea cucumber was cleaned after gutting, thoroughly crushed by a beater (JR05-300, SUPOR, Hangzhou, China), hydrolyzed with a complex protease (papain: trypsin = 2:1, *w*/*w*, Novozymes), precipitated, filtrated, and spray dried to obtain the SCH.

### 2.3. Determination of the Basic Constituents of SCH

The moisture content of SCH was measured by using the direct drying method. Briefly, 2−10 g of SCH powder was accurately weighed, dried at 101–105 °C for 4 h, and weighed after a constant weight was achieved. The ash content of SCH was measured by using the high−temperature ashing method, wherein, the SCH powder was accurately weighed, carbonized to smokeless at a high temperature on an induction furnace, burned in a Muffle furnace at 550 ± 25 °C for 4 h, and weighed after a constant weight was achieved. The total protein content of SCH was determined by the Kjeldahl method. SCH powder was accurately weighed and digested in the digestion tube, which was continued for 1 h after the temperature of the digestion furnace reached 420 °C. The digestion process was stopped when the liquid turned green and transparent. After the digestion tube was cooled, the total protein content of SCH was measured on the automatic Kjeldahl nitrogen analyzer (K9840, Haineng, China).

### 2.4. Determination of the Amino Acid Composition of SCH

The amino acid composition of SCH was determined by using an amino acid analyzer (L-8900, Hitachi, Japan) as described elsewhere [19]. Briefly, 15 mg of the protein samples and 10 mL of 6 M HCl were added to a hydrolysis tube and heated at 110 °C for 24 h. The dried hydrolysate was then dissolved in 0.02 M HCl and filtered and tested on the machine.

### 2.5. Identification of Oligopeptides in SCH by LC-MS

SCH was prepared into a solution using a certain concentration and centrifuged in an ultrafiltration tube with an interception capacity of 3000 Da. Next, filtrate < 3000 Da was collected for oligopeptide analysis. The peptide was sequenced by liquid chromatography−mass spectrometry (LC−MS) as per the standard method, albeit with some modifications [20]. Oligopeptides were identified by ultra-performance liquid chromatography (Dionex Ultimate 3000, Waltham, MA, USA) and mass spectrometry (Thermo Scientific Q active, Waltham, MA, USA). The peptides were then separated into a column (accucore C18, 100 × 2.1 mm^2^, 2.6 μm). The mobile phase A was composed of ultrapure water (containing 0.1% formic acid) and the mobile phase B was composed of acetonitrile. The linear elution gradient of the mobile phase B was 5–70%. Maxquant software was used for peptide matching. The measured oligopeptides were evaluated by PeptideRanker (http://distilldeep.ucd.ie/PeptideRanker/, accessed on 10 December 2022).

### 2.6. Animals and Experimental Design

All animal experiments were conducted in accordance with the Experimental Animal Care and Use Guidelines of Tianjin University of Science and Technology and were approved by the Animal Ethics Committee of Tianjin University of Science and Technology (approval number: TUST20210907). Fifty female Balb/c mice (age: 6−8 weeks) were purchased from Beijing Sipeifu Biotechnology Co., Ltd. (Beijing, China), and fed adaptively a week before the experiment. The mice were randomly assigned to the normal group, model group (Cy, 80 mg/kg·BW), and SCH groups (200, 500, and 1000 mg/kg·BW). The mice in the normal and model groups were administered 0.2 mL of Sterile water, once a day. The SCH groups received the same volume of SCH solution (200, 500, and 1000 mg/kg·BW) for 34 days, respectively. On days 27, 28, and 29, Cy was injected intraperitoneally, except for the normal group, to establish an immunosuppressive model. After the experiment, the thymus and spleen of the mice were extracted aseptically and weighed. A part of the small intestine and colon tissue was preserved in paraformaldehyde for histopathological observation, and a part of the small intestine tissues was stored in a −80 °C refrigerator for Western blotting. The colonic contents of the mice were collected and stored in a −80 °C refrigerator for detection of the fecal gut microbiota.

### 2.7. Immune Organ Index

At the end of the experiment, the thymus and spleen of the mice were removed and weighed aseptically. The immune organ index was calculated using the following formula:

Index (mg/g) = immune organ weight (mg)/body weight (g)

### 2.8. Detection of Biochemical Indexes

The content of serum ALT, AST, IgG, and small intestinal sIgA was measured using the test kit according to the manufacturer’s instructions. The absorbance was read with a Thermo Multiscan FC microplate. The whole blood was centrifuged at 4 °C to obtain mouse serum, and the intestinal tissue was homogenized on ice with a glass homogenizer to obtain intestinal tissue homogenate.

### 2.9. Histopathological Examination

Mice’s small intestine and colon tissues were preserved with paraformaldehyde, paraffin−embedded and sliced, and stained with hematoxylin and eosin (H&E).

### 2.10. Western Blotting

The mouse small intestine tissues were cleaned with cold phosphate buffer (PBS) and then grounded thoroughly on an ice bath with RIPA buffer containing protease inhibitor (Solarbio, Beijing, China). The supernatant was obtained after centrifugation of the mouse small intestine homogenate at 12,000× *g* at 4 °C for 5 min. The protein concentration of the small intestine homogenate was determined by using a total protein quantification kit (Jiancheng, Nanjing, China). The small intestine homogenate was mixed with the loading buffer (Biosharp, Hefei, China) and boiled for 5 min. The proteins were isolated by 12% SDS/PAGE and transferred onto a PVDF membrane. The membrane was treated with 5% skim milk for 1 h and incubated with primary antibodies at 4 °C overnight, followed by treatment with a secondary antibody at room temperature for 1 h. The remaining antibodies on the PVDF membrane were washed with the TBST solution. The protein was detected by using a chemiluminescent substrate assay kit (Biosharp, Hefei, China), and the protein band blotting was performed using a Chemiluminescence imager (LAS4000, GE, Boston, MA, USA). The protein band strength was detected by Image J software.

### 2.11. Gut Microbiota Analysis

Genomic DNA was extracted from the colonic contents of mice in each group, and the concentration and purity of genomic DNA were detected by agarose gel electrophoresis. Genomic DNA was diluted in sterile water and used as a template for PCR amplification. The high variable region of 16S rDNA gene V3–V4 was used as a primer for amplification, and the PCR products were recovered and purified. After the library was constructed, NovaSeq 6000 was used for sequencing.

### 2.12. Statistical Analysis

All experiments were statistically analyzed using GraphPad Prism 8, Origin 9, and SPSS19. The results were expressed as the mean ± SD (X ± SD). The statistical significance between the two groups was calculated by using Student’s *t*-test and that among multiple groups was determined by one-way analysis of variance (ANOVA) followed by Tukey’s test. *p* < 0.05 was considered to indicate statistical significance, and the significance level was set at * *p* < 0.05 and ** *p* < 0.01.

## 3. Results

### 3.1. Amino Acid Composition and Oligopeptide Sequence Analysis of SCH

**Figure 1:**
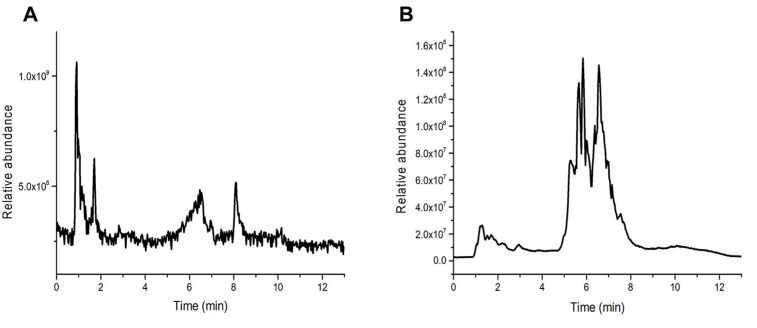
Total ion current diagram of SCH. Scanning range: 50–500 *m*/*z* (**A**) and 500–2000 *m*/*z* (**B**).

The protein content, moisture content, and ash content of SCH were 94.71 ± 4.69%, 2.95 ± 0.11%, and 0.05 ± 0.00%, respectively. In this experiment, LC−MS was used to obtain the total ion flow diagram of SCH (Figure 1). The amino acid composition and oligopeptide sequence identification results of SCH are shown in Table 1 and Table 2, respectively. A total of 16 amino acids were detected in SCH, and the contents of Gly, Glu, Ala, Pro, and Asp were higher (Table 1). The oligopeptides detected in SCH were input to PeptideRanker for activity evaluation. The activity score of peptides in Table 2 were all >0.5. The oligopeptides with high activity in SCH ranged in molecular weight from 189.12−547.27 *m*/*z* and were mainly composed of oligopeptides with 2−4 amino acids.

### 3.2. Effect of SCH on the Immune Organ Index in Mice

**Figure 2:**
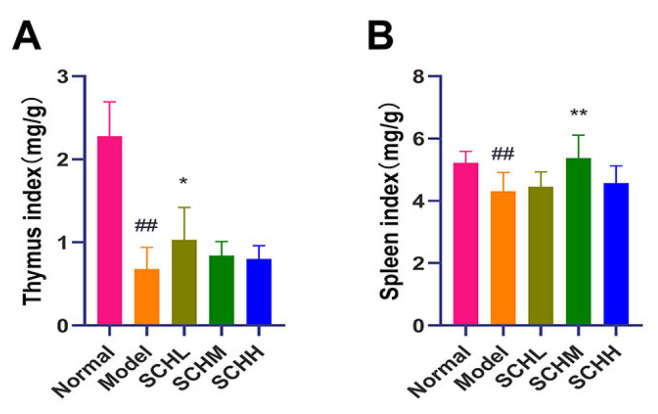
Effects of SCH on the thymus index (**A**) and spleen index (**B**) in immunosuppressed mice. The results are presented as the means ± SD (n = 10). ## *p* < 0.01 compared with the normal group, * *p* < 0.05, ** *p* < 0.01 when compared with the model group.

In this study, the mice were intraperitoneally injected with 80 mg/kg BW Cy for three days to establish an immunosuppressive model. The immune organ index directly reflected the immune state of the body [14]. The effect of SCH on the immune organ index is shown in Figure 2A,B. The thymus index and spleen index of the model group were significantly lower than that of the normal group (*p* < 0.01). When compared with the model group, the thymus index in the SCHL group was significantly increased (*p* < 0.05) and the spleen index in the SCHM group was significantly increased (*p* < 0.01). These results suggested that SCH could help restore the thymus and spleen indexes in immunosuppressed mice, thereby restoring their immunomodulatory abilities.

### 3.3. Effects of SCH on the Biochemical Indices in Mice

**Figure 3:**
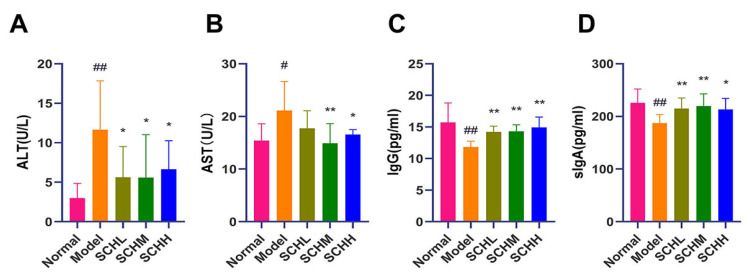
Effects of SCH on serum ALT (**A**), AST (**B**), IgG (**C**), and small intestinal sIgA (**D**) levels in immunosuppressed mice. The results are presented as the means ± SD (n = 10). # *p* < 0.05, ## *p* < 0.01 compared with the normal group, * *p* < 0.05, ** *p* < 0.01 when compared with the model group.

To assess the effect of SCH on immunosuppressed mice, the serum ALT, AST, IgG, and small intestinal sIgA levels were measured (Figure 3A−D). When compared with the model group, the ALT levels in the SCH groups were significantly decreased (*p* < 0.05) and the AST level in the SCHM group was significantly decreased (*p* < 0.01). When compared with the model group, the IgG levels of mice in the SCH groups were significantly increased (*p* < 0.01) and the sIgA levels in the SCHL and SCHM groups were significantly increased (*p* < 0.01). In conclusion, SCH can reduce liver injury caused by Cy and improve immunity by increasing the levels of immunoglobulins.

### 3.4. Effect of SCH on Histological Changes of the Small Intestine and Colon

**Figure 4:**
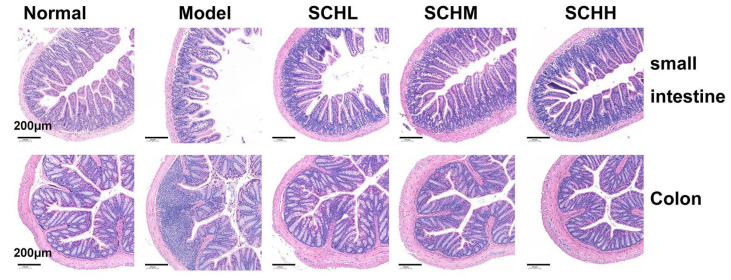
Effect of SCH on the morphology of the small intestine and colon in immunosuppressed mice (n ≥ 3).

To observe the effect of SCH on intestinal morphology, small intestine, and colonic tissues were collected for histopathological analysis (Figure 4). H&E staining revealed that the small intestine and colon in the normal group had normal histology and an intact surface structure. In the model group, the villi of the small intestine became shorter and slightly swollen and the depression depth decreased. The model mice group showed severe colon injury. When compared with the model group, the damage to the small intestine and colon was relieved in the SCH groups. The results revealed that Cy damaged the surface structure of the intestinal tissues and SCH improved the intestinal mucosal damage in immunosuppressed mice.

### 3.5. Effect of SCH on the Expression of NF-κB Pathway Proteins in Mice

**Figure 5:**
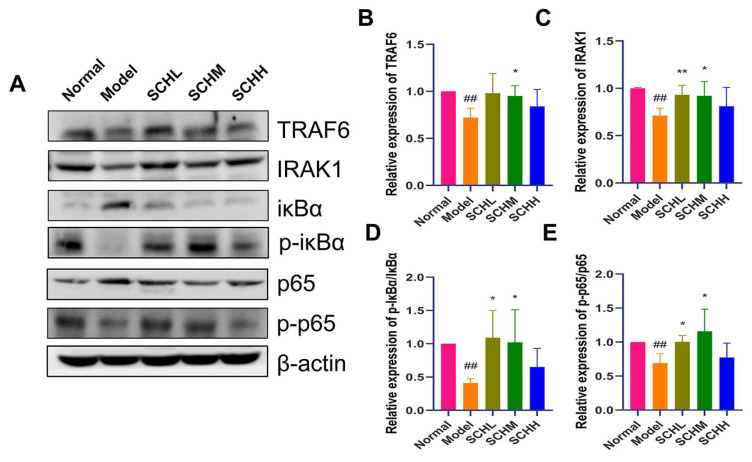
The effect of SCH on the relative expression levels of TRAF6 (**B**), IRAK1 (**C**), p−iκB−α/iκB−α (**D**), and p−p65/p65 (**E**) in immunosuppressed mice and the image of the above protein bands (**A**). The results are presented as the means ± SD (n ≥ 3). ## *p* < 0.01 compared with the normal group, * *p* < 0.05, ** *p* < 0.01 when compared with the model group.

The expression level of the NF−κB pathway-related proteins in the small intestine of mice in each group was detected by Western blotting. Figure 5A depicts the bands of each protein. As shown in Figure 5B,C, when compared with the normal group, the protein expression levels of TRAF6 and IRAK1 in the model group were significantly reduced (*p* < 0.01). When compared with the model group, the expression level of TRAF6 protein in the SCHM group was significantly increased (*p* < 0.05), while the expression level of IRAK1 protein in the SCHL group was significantly increased (*p* < 0.01). As shown in Figure 5D,E, the phosphorylation levels of IκBα and P65 in the model group were significantly reduced when compared with those in the normal group (*p* < 0.01). When compared with the model group, the phosphorylation levels of IκBα and P65 in the SCHL and SCHM groups were significantly increased (*p* < 0.05).

### 3.6. Effect of SCH on the Mice Gut Microbiota

**Figure 6:**
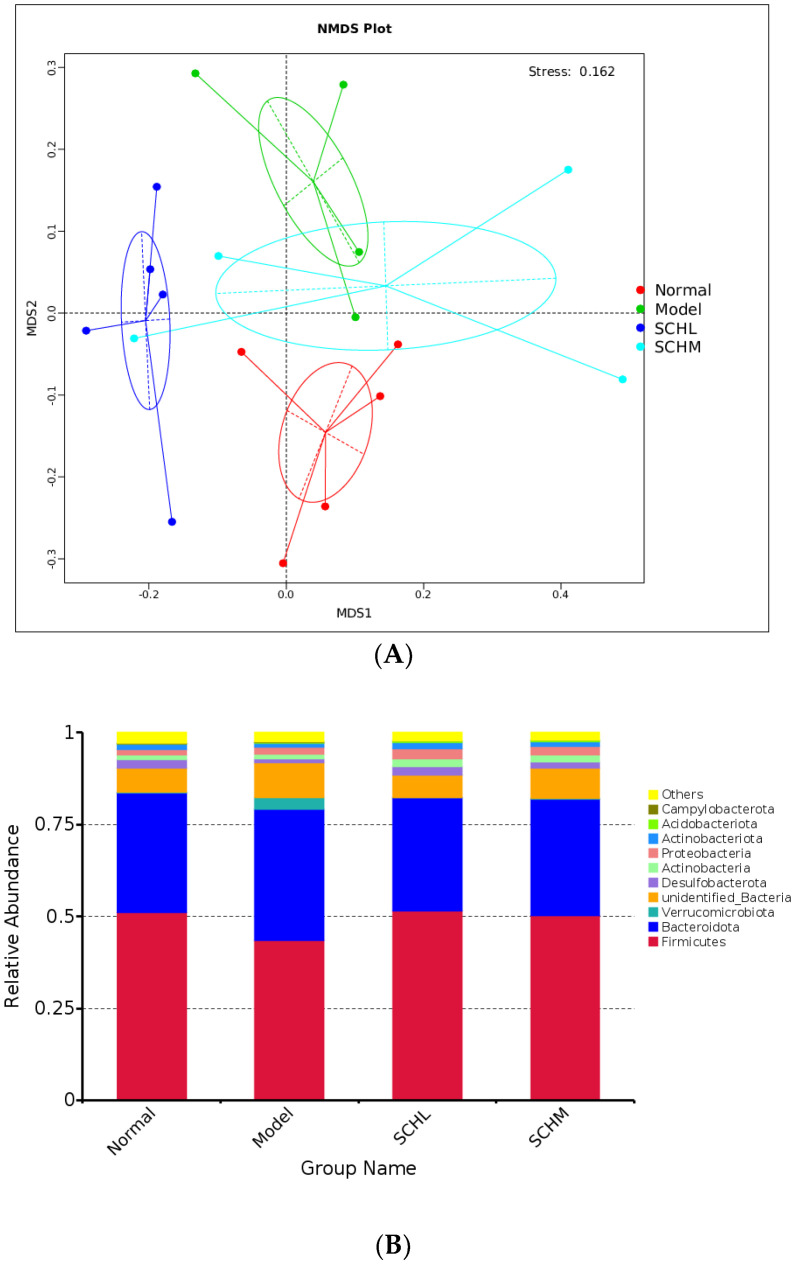

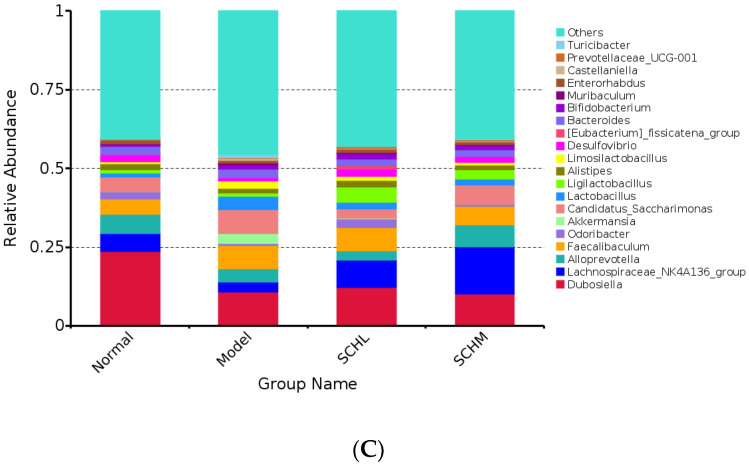
Effect of SCH on gut microbiota in immunosuppressed mice. NMDS analysis of the gut microbiota in each group (**A**). The relative abundance of gut microbiota of mice in each group at the phylum (**B**) and genus (**C**) levels. The results are presented as the means ± SD (n ≥ 4).

**Figure 7:**
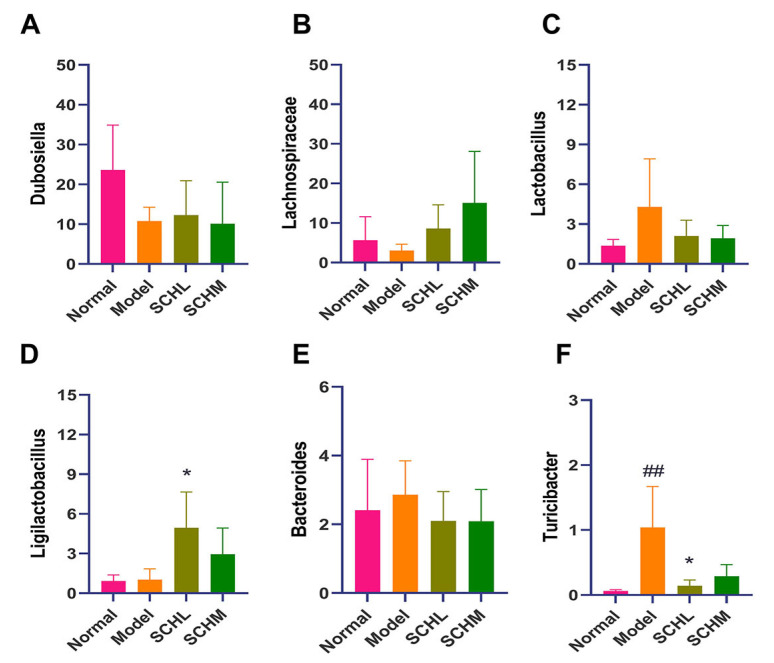
Effect of SCH on the gut microbiota in immunosuppressed mice. The relative abundance of *Dubosiella* (**A**), *Lachnospiraceae* (**B**), *Lactobacillus* (**C**), *Ligilactobacillus* (**D**), *Bacteroides* (**E**), and *Turicibacter* (**F**) in each group. The results are presented as the means ± SD (n ≥ 4). The results are presented as the means ± SD, ## *p* < 0.01 compared with the normal group, * *p* < 0.05 when compared with the model group.

The colonic contents of mice were collected and 16S rDNA sequencing was performed to analyze the effect of SCH on the gut microbiota of mice. Because SCHL and SCHM groups had a more significant immunity enhancement effect in the above−mentioned tests, we selected the colonic contents of mice in the normal group, model group, SCHL group, and SCHM group for gut microbiota sequencing. Non−metric Multi−Dimensional Scaling (NMDS) was performed to describe the correlations between the groups of microbiomes (Figure 6A). When compared with the normal group, the gut microbiota composition of Cy mice changed after modeling. In NMDS analysis, the model group was completely separated from the normal group, the SCHL group was completely separated from the other groups, and the SCHM group was distributed between the normal and model groups. Therefore, Cy induced changes in the gut microbiota composition of mice, and SCH intervention made the gut microbiota composition of immunosuppressed mice approximate to that of the normal mice. To further determine the changes in the gut microbiota after SCH intervention, the bacterial composition was compared at the phylum and genus levels in the normal, model, SCHL, and SCHM groups (Figure 6B,C and Figure 7A−F). At the phylum level, *Bacteroidetes* and *Firmicutes* were the main phyla identified, and it was noted that the abundance of *Bacteroidetes* increased while the abundance of *Firmicutes* decreased in the model group. However, SCH intervention reversed this trend. At the genus level, the abundance of *Dubosiella* and *Lachnospiraceae* decreased in the model group and increased in the SCH groups, while the abundance of *Lactobacillus*, *Bacteroides*, and *Turicibacter* increased in the model group, but decreased in the SCH group. The relative abundance of *Ligilactobacillus* in the SCHL and SCHM groups was higher than that in the model group.

## 4. Discussion

Sea cucumber is a type of seafood with a high edible and medicinal value, with the characteristics of high protein and low−fat [21]. Sea cucumber peptides have been proven to possess biological activities such as antioxidation, improving immunity, enhancing memory, and reducing blood sugar. Bioactive peptides are easy to digest, can play a variety of physiological functions in the human body, and generally exhibit higher biological activity than their parent proteins [1]. Cyclophosphamide (Cy), as a chemotherapy drug, has an immunosuppressive effect, which can destroy the gastrointestinal mucosal barrier and induce an imbalance in the gut microbiota [14]. Past studies have demonstrated that the immunomodulatory ability of peptides depends on their amino acid composition, sequence, length, charge, hydrophobicity and structure [1]. The amino acids in sea cucumber are rich, including all types of essential amino acids and non−essential amino acids, and have a high content of amino acids such as glycine, glutamic acid, proline, and alanine [22]. Similarly, the amino acid composition analysis of SCH in this study revealed a high content of glycine, glutamic acid, alanine, and proline. By analyzing the oligopeptides of SCH, 26 oligopeptides with high activity scores were determined. Therefore, we investigated the immune−enhancing ability of SCH by using a Cy−induced immunosuppression mice model.

The thymus and spleen are both important immune organs of the body, and the organ index reflects the immune state of the body [23]. SCH can improve the immune organ index of immunosuppressed mice. Cy has liver−damaging effects [24]. ALT and AST are both indexes of liver injury, and they were both decreased in the SCH groups, indicating that SCH could alleviate liver injury caused by Cy. IgG and sIgA are important immunoglobulins in the body. SCH can effectively increase the IgG and sIgA levels of immunosuppressed mice, implying that SCH can improve immunity by increasing the level of immunoglobulins.

The intestinal mucosa is resistant to food contamination, chemicals, and pathogens and plays an important role in immune regulation [25]. Intestinal mucosa acts as a barrier against the invasion of pathogenic microorganisms. The integrity of intestinal morphology determines whether the intestinal barrier can function normally. SCH can reduce intestinal mucosal damage and enhance intestinal mucosal integrity in immunosuppressed mice.

NF−κB is a nuclear transcription factor that regulates the expression of a large number of genes essential for the regulation of host immunity [24]. NF−κB is activated by complex molecular interactions with the adaptor proteins, phosphorylation, and ubiquitase, to regulate gene expression [26]. TRAF6 is a key regulator of the activation of transcription factor NF-κB [27]. IRAKs are serine/threonine kinases that play a key role in innate immune responses [28]. Both TRAF6 and IRAK1 are upstream proteins of the NF−κB pathway. It has been reported that co−fermented collagen peptide−pineapple juice and barley young leaf polysaccharide both alleviate Cy−induced immunosuppression by activating the NF−κB pathway [12,29]. In this study, the expression levels of TRAF6 and IRAK1 protein and the phosphorylation levels of Iκbα and p65 in immunosuppressed mice were increased after SCH intervention, suggesting that SCH could activate the NF-κB pathway and improve the immunity of mice.

Gut microbiota is closely related to host growth, immune regulation, and intestinal health [30]. *Bacteroidetes* and *Firmicutes* are two important dominant phyla among the gut microbiota, and their ratio has been associated with obesity and inflammation [31]. In addition, the gut microbiota of Cy−induced immunosuppressed mice has increased *Bacteroidetes* and decreased *Firmicutes*; therefore, sodium alginate intervention can improve immunity by alleviating the changes [14]. Similarly, our study findings demonstrated that SCH decreased the abundance of *Bacteroidetes* and increased the abundance of *Firmicutes*. Dubosiella newyorkensis has an anti−aging function, and it may be superior to resveratrol in reducing oxidative stress, improving vascular endothelial functions, and regulating the gut microbiota [32]. Past studies have demonstrated that *Dubosiella* may help relieve colitis [33]. *Lachnospiraceae* can produce a short−chain fatty acid, and its abundance has been positively correlated with the expression of tight junction proteins [34,35]. *Ligilactobacillus salivarius* is a lactobacillus with beneficial functional properties such as antibacterial activity, immunity, and the ability to regulate gut microbiota [36]. It has been shown that Chinese and Brazilian propolis can relieve DSS−induced colitis, and propolis has been reported to reduce the relative abundance of *Bacteroides*, which is important for maintaining intestinal hemostasis [37]. Urtica dioica reduces diet−induced weight gain and insulin resistance, which is associated with decreased *Turicibacter* proliferation and altered amino acid metabolism [38]. The addition of caffeic acid decreased the relative abundance of *Bacteroides* and *Turicibacter*, thereby altering the composition of the gut microbiota and improving colitis [39]. In this study, when compared with the model group, the relative abundance of *Dubosiella*, *Lachnospiraceae*, and *Ligilactobacillus* in the SCH groups increased, while the abundance of *Lactobacillus*, *Bacteroides*, and *Turicibacter* decreased. The above results indicate that SCH has a good effect on the gut microbiota of Cy induced immunosuppressive mice, which can alter the structure of the gut microbiota and the relative abundance of specific microbiota.

## 5. Conclusions

In this study, Cy was used to establish an immunosuppression model in mice, and the effect of SCH on the immunosuppressed mice was explored. The results suggested that SCH could increase the indexes of the thymus and spleen, decrease the levels of serum ALT and AST, increase the contents of serum IgG and small intestinal sIgA, and reduce the damage to the small intestine and colon. SCH also activated the NF-κB pathway by increasing the expression levels of TRAF6 and IRAK1 proteins and increasing the phosphorylation of IκBα and P65, thereby enhancing immunity. In addition, SCH regulated the intestinal health of mice by increasing the relative abundance of *Dubosiella*, *Lachnospiraceae*, and *Ligilactobacillus* and decreasing that of *Lactobacillus*, *Bacteroides*, and *Turicibacter,* thereby alleviating the imbalance of gut microbiota caused by Cy. In this study, SCH has a good effect on Cy−induced immunosuppression mice, which is worth developing as a functional food and provides a new idea for exploring the molecular mechanism of bioactive peptides to enhance immunity.

## Figures and Tables

**Table 1:** Contents of amino acids in SCH.

Amino Acid	Content (%)
Gly	7.77 ± 0.07
Glu	6.49 ± 0.19
Ala	3.81 ± 0.07
Pro	3.41 ± 0.07
Asp	3.25 ± 0.10
Arg	2.92 ± 0.05
Thr	1.67 ± 0.04
Val	1.58 ± 0.04
Phe	1.47 ± 0.15
Ser	1.47 ± 0.06
Leu	1.34 ± 0.10
Lys	1.04 ± 0.19
Tyr	0.86 ± 0.07
His	0.64 ± 0.22
Ile	0.62 ± 0.03
Met	0.58 ± 0.02

**Table 2:** Determination of the SCH oligopeptide sequence by LC–MS.

Number	Observed Mass (*m*/*z*)	Calculated Mass (*m*/*z*)	Charges	Mass Error	RT (min)	Intensity	Activity Prediction Score	Sequence
1	297.12674	296.11946	1	−2.0494	6.77	1,906,100	1.00	MF
2	223.10772	222.10044	1	−1.7651	3.65	72,543,000	0.99	GF
3	547.27758	546.2703	1	3.2459	6.04	96,065	0.99	WRW
4	161.59732	321.18009	1;2	−0.9894	1.49	27,211,000	0.99	FR
5	181.10277	360.19099	2	−1.2965	2.09	1,190,500	0.98	WR
6	539.23948	538.2322	1	1.6862	1.32	144,310	0.97	GFRC
7	207.07979	206.07251	1	−3.0502	1.52	23,017,000	0.95	GM
8	279.17032	278.16304	1	−1.1567	7.26	12,614,000	0.95	IF
9	294.14483	293.13756	1	−1.9427	2.32	2,390,400	0.92	FQ
10	173.09207	172.08479	1	−1.468	1.13	8,256,900	0.91	GP
11	165.10023	328.1859	2	−1.0154	1.01	3,343,200	0.84	PGR
12	548.27218	547.2649	1	−1.3542	1.97	18,687	0.82	GCRR
13	232.14042	231.13314	1	−1.7049	1.01	51,228,000	0.77	GR
14	239.10263	238.09536	1	−1.3903	2.08	17,041,000	0.74	GY
15	279.13393	278.12666	1	2.1445	2.52	1,674,600	0.74	PY
16	505.33845	504.33117	1	−0.073093	6.8	4,023,000	0.70	IIIF
17	286.17613	285.16886	1	−2.1445	5.87	7,544,800	0.69	IGP
18	194.6244	387.23425	2	−1.0358	1.01	6,610,400	0.64	RGR
19	237.09035	236.08308	1	−1.4734	1.46	9,146,500	0.63	SM
20	180.10551	358.19647	2	−1.6217	1.11	11,547,000	0.62	SPR
21	295.12885	294.12157	1	−4.0948	3.16	3,685,100	0.59	FE
22	173.11588	344.2172	2	−0.90103	1.34	12,308,000	0.57	GIR
23	166.11367	330.21279	2	0.50389	1.01	2,633,900	0.57	RR
24	152.0924	302.17025	2	−1.5916	1.12	77,656,000	0.55	GAR
25	519.22316	518.21588	1	2.0725	5.12	352,480	0.54	NCPK
26	189.12337	188.11609	1	−1.1305	1.37	45,416,000	0.50	IG

## Data Availability

The data presented in this study are available on request from the corresponding author.
